# Real-world comparison of terlipressin vs. octreotide as an adjuvant treatment in the management of variceal bleeding

**DOI:** 10.1038/s41598-024-56873-x

**Published:** 2024-03-20

**Authors:** H. Rehman, S. T. Rehman, S. Zulfiqar, S. Awan, Shahab Abid

**Affiliations:** Section of Gastroenterology, Department of Medicine, Aga Khan University, National Stadium Road, P.O Box 3500, Karachi, Pakistan

**Keywords:** Gastrointestinal diseases, Gastrointestinal system, Hepatology

## Abstract

Variceal bleeding is a major complication and the leading cause of death in patients with cirrhosis and portal hypertension. This study aims to compare the efficacy and safety of terlipressin vs octreotide as an adjuvant to endoscopic management of patients with esophageal variceal bleeding in a real-time scenario. We reviewed the medical records of patients with esophageal variceal bleeding from January 2005 to December 2020 at our tertiary care Aga Khan University Hospital. Mortality was assessed after 6 weeks. A total of 842 patients with variceal bleed were evaluated. 624 patients (74.1%) and 218 patients (25.9%) received Terlipressin and Octreotide respectively. On multiple regression analysis, cardiac events during hospital stay (OR: 11.22), presence of Porto-systemic encephalopathy (OR: 3.79), and elevated bilirubin levels at the time of presentation were found to be independent risk factors for increased six weeks mortality. Moreover, cardiac events during hospital stay (OR: 3.26), Porto-systemic encephalopathy at presentation (OR: 3.06), and octreotide administration (OR: 1.80) were identified as independent risk factors for increased length of hospital stay. Terlipressin and Octreotide have similar outcomes in terms of control of bleeding, hospital stay, mortality, and side effects when used as adjuvant therapy for the management of variceal bleeding.

## Introduction

Variceal bleeding is a major complication and the leading cause of death in patients with cirrhosis and portal hypertension. A yearly prevalence of 5–15% of variceal bleeding has been reported in cirrhotic patients, with approximately 70% of all upper gastrointestinal bleeding (UGIB) episodes being attributed to variceal bleeding^1,2^. Despite advancements in endoscopic and pharmacological management, it is still associated with a mortality of 15–20% in advanced cirrhosis^3^. The mortality is higher in patients in whom the acute bleeding episode cannot be controlled initially, or who develop rebleeding within 5 days^4^.

Medical therapy for esophageal variceal bleeding (EVB) is aimed at reducing the splanchnic blood flow and portal pressure. Endoscopic esophageal band ligation is the preferred procedure for achieving hemostasis, and if it proves to be technically difficult, endoscopic injection sclerotherapy (EIS) can be performed instead. If variceal bleeding is suspected, initial resuscitation is done with either colloids or crystalloids for volume correction. Vasoactive agents are administered promptly to reduce the portal blood flow and are ideally maintained for 2–5 days.

The most common vasoactive agents used include terlipressin, vasopressin, somatostatin, and octreotide. Terlipressin, a potent vasoconstrictor, is a long-acting synthetic derivative of vasopressin, with fewer adverse effects. It predominantly acts on the V1 receptors present in the arterial smooth muscle within the splanchnic circulation, resulting in decreased blood flow and portal pressure. Somatostatin and its analogs (octreotide, vapreotide) induce selective splanchnic vasoconstriction by inhibiting the release of vasodilator glucagon and through blunting of postprandial splanchnic hyperemia^5^. Hence, it decreases the portal blood flow, with minimal systemic side effects.

Multiple studies have reported no change in the mortality of patients who get treated with terlipressin or octreotide as an adjunct to esophageal therapy^6^. Terlipressin and somatostatin both were found to have equal effects on 30-day mortality in cirrhotic patients with EVB and renal functional impairment^7^. A meta-analysis conducted in 2018 by Zhou et al. reported that terlipressin was found to be significantly inferior to octreotide in achieving control of bleeding within 24 h, however, there was no significant difference in the in-hospital mortality^3^.

The primary objective of this study is to compare the efficacy and safety of terlipressin vs octreotide as an adjuvant to endoscopic management of patients with variceal bleeding in a real-time scenario. Besides the comparison of the two treatment groups, this study also aims to identify individual risk factors that contribute to increased mortality or increased length of hospital stay.

## Methods

### Study design and settings

In this single-center study, we reviewed the medical records of all patients who were admitted for the control of upper GI bleeding. A total of 2190 records were screened and finally, 842 were recruited for analysis for the period of January 2005 to December 2020 at The Aga Khan University Hospital.

### Inclusion criteria

We included all the patients with cirrhosis who presented to the emergency room of our hospital with an upper gastrointestinal bleed and were diagnosed as a case of variceal bleeding.

### Exclusion criteria

Patients who were diagnosed with non-variceal bleeding on endoscopy, or those with gastric variceal/portal hypertensive gastropathy-related bleed were excluded from this review. We did not include patients who underwent endoscopy after a delay of > 24 h due to any reason. Patients who did not receive Terlipressin or Octreotide as a treatment were excluded. Patients having advanced cardiovascular or pulmonary diseases were also excluded.

### Ethics clearance

Ethical approval was obtained from the Ethical Research Committee at The Aga Khan University. The need for informed consent was waived by The Aga Khan University- Ethical Research Committee as this is a retrospective study of medical records. Extra precaution was taken to ensure total anonymity of the data during its access and extrapolation, analysis, stratification, and eventual presentation in this manuscript. All methods were performed in accordance with standardized guidelines and regulations.

### Data collection

Data related to the demographics, etiology of hepatitis, and Child Class was extracted. The presence of portal-systemic encephalopathy at the time of presentation along with the vitals was recorded. Basic labs including platelet count, serum creatinine, SGPT, total bilirubin, serum albumin, GGT, and prothrombin time were also recorded from the database.

**Figure 1:**
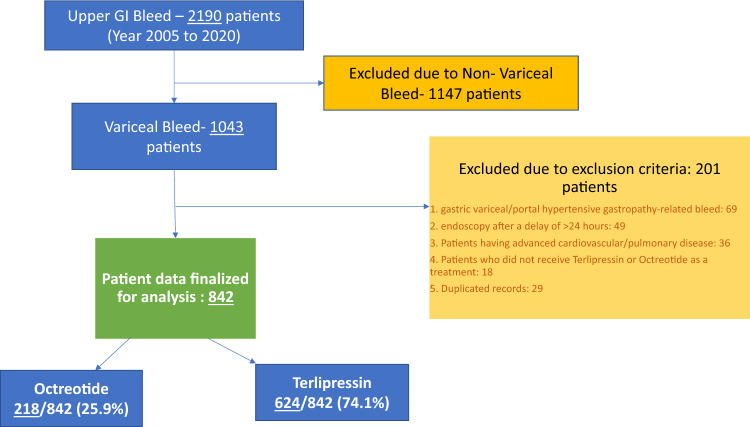
Flow chart representing the methodology of stratifying data in this cohort.

Endoscopic therapy was used in most of the patients after diagnosis. After stabilizing the patient hemodynamically, they underwent endoscopy, and either ethanolamine oleate was injected para-variceal or esophageal variceal band ligation (EVBL) was done. The patients were treated according to the standard protocol for terlipressin or octreotide administration before endoscopic therapy. We stratified the data in this cohort as explained in our flowchart ** (**Fig. 1**)**.

The total duration of study treatment was 6 weeks per subject. Terlipressin was administered at a dose of 2 mg by IV bolus followed by 1 mg IV every 6 h. Octreotide was administered at 100 ml bolus of 100 µg IV octreotide prepared as 1 µg octreotide in 1 ml of 0.45% dextrose saline. The duration of the medicines for both agents was for 72 h followed by 50 μg/h IV infusion up to 72 h.

The assessment was done in the hospital for the control of variceal bleeding and mortality was assessed after 6 weeks. The Baveno V criteria and definitions were used for the assessment of failure to control bleeding. According to the criteria, bleeding control should be achieved within 120 h (5 days) and failure was defined as death or the need to change therapy if any of the following occurred^8^.

1. Fresh hematemesis or nasogastric aspiration of ≥ 100 mL of fresh blood ≥ 2 h after the start of a specific drug treatment or therapeutic endoscopy.
2. Development of hypovolemic shock
3. A drop of 3 g in hemoglobin levels within any 24 h if no transfusion is administered
4. The potential value of an index of blood transfusion requires prospective validation [e.g., the adjusted blood requirement index (ABRI) from Baveno IV].

### Statistical analysis

The analysis was performed using SPSS (Statistical Package of Social Sciences) version 19. Continuous variables with normal and non-normal distributions were reported as mean ± SD and median [inter-quartile range (IQR)], respectively. Prevalence (%) of demographic as well as clinical factors was assessed and stratified by treatment arms. Continuous variables with normal distribution were analyzed using an independent samples t-test, while those with skewed distribution were analyzed using the Mann–Whitney U test. Multivariate logistic regression analysis was used to identify risk factors of hospital stay and mortality. All p-values were based on two-sided tests and significance was set at a p-value less than 0.05.

## Results

### Demographics and patients’ characteristics

A total of 842 participants were enrolled in the study. The mean age was 51.9 years and there were 576/842 (68.7%) males. A total of 624/842 (74.1%) patients received terlipressin whereas 218/842 (25.9%) received Octreotide as adjuvant therapy in the management of variceal bleeding.

The most common underlying etiology for portal hypertension and esophageal varices in both the treatment groups was chronic hepatitis C virus infection (52.1%), followed by non-B, non-C, and chronic hepatitis B. Alcoholic hepatitis was found to be present in a very small subset of the total study population (4.3%). Refer to Table 1 for the etiology of chronic liver disease in the octreotide and terlipressin groups.

**Table 1:** Baseline characteristics of the study population (n = 842).

	Total	Terlipressin; n = 624	Octreotide; n = 218	*p* value
Age, years	51.9 ± 12.7	50.4 ± 12.2	55.8 ± 13.1	< 0.001
Median (IQR); range	53 (45–60); 18–95			
Sex				
Male	576 (68.7)	429 (68.8)	150 (68.8)	0.98
Female	263 (31.3)	195 (31.3)	68 (31.2)	
Etiology				
HBV	46 (5.5)	37 (5.9)	9 (4.1)	0.08
HCV	439 (52.1)	324 (51.9)	115 (52.8)	
HBV & HCV	31 (3.7)	26 (4.2)	5 (2.3)	
NBNC	161 (19.1)	114 (18.3)	47 (21.6)	
Alcoholic	36 (4.3)	28 (4.5)	8 (3.7)	
HCV & alcoholic	10 (1.2)	8 (1.3)	2 (0.9)	
HBV & HDV	3 (0.4)	0	3 (1.4)	
Others	116 (13.8)	87 (13.9)	29 (13.3)	
Child class				
A	175 (34.4)	157 (37.3)	18 (20.5)	< 0.001
B	39 (7.7)	25 (5.9)	14 (15.9)	
C	295 (58.0)	239 (56.8)	56 (63.6)	
PSE at presentation	161 (19.1)	115 (18.4)	46 (21.1)	0.38
Hb at presentation (g/dl)	8.7 ± 2.0	8.8 ± 2.0	8.4 ± 2.2	0.03
Pulse in ER (beats/min)	97.0 ± 19.3	96.8 ± 19.4	97.6 ± 19.1	0.61
Oxygen saturation %	98.0 ± 2.8	98.0 ± 3.0	97.8 ± 2.3	0.3
Systolic BP in ER (mmHg)	117.6 ± 20.7	117.7 ± 20.2	117.0 ± 22	0.67
Diastolic BP in ER (mmHg)	67.6 ± 13.4	68.1 ± 13.2	66.1 ± 13.7	0.06
Platelet’s count	125.8 ± 73.1	119.6 ± 70.0	142.5 ± 78.8	< 0.001
Serum creatinine (mg/dl)	1.2 ± 0.9	1.2 ± 0.9	1.3 ± 0.8	0.12

Median (IQR).

The majority of the patients belonged to Child Class C, followed by Child A and Child B respectively. A higher proportion of patients with Child Class C were given Terlipressin (239/295, 81%). Both groups were comparable in terms of hemodynamic parameters in the ER.

### Comparison between terlipressin and Octreotide in morbidity and mortality

There was no significant difference in the length of hospital stay, the number of packed cells transfused, control of variceal bleed after endoscopy, cardiac effects during hospital stay, and mortality after 6 weeks between the two treatment groups as shown in Table 2. The median length of hospital stay averaged at 4.81 in terlipressin with the highest stay being 29 days and the lowest being 1 day. Octreotide median stay numbers were noted as average stay being 5.21 days, highest stay being 15 and lowest being 1.

**Table 2:** Comparison between terlipressin and octreotide in mortality and morbidity.

	Terlipressin; n = 624	Octreotide; n = 218	*p* value
Control of variceal bleeding after EGD	556 (89.1)	191 (87.6)	0.55
No. of packed cells transfusion	3.0 ± 3.4	3.2 ± 2.7	0.057*
Length of hospital stay	4.8 ± 2.8	5.2 ± 2.6	0.057
Mortality	37 (5.9)	15 (6.9)	0.61
Cardiac effects during hospital stay	20 (3.2)	11 (5.0)	0.21

### Factors associated with prolonged hospital stay

**Figure 2:**
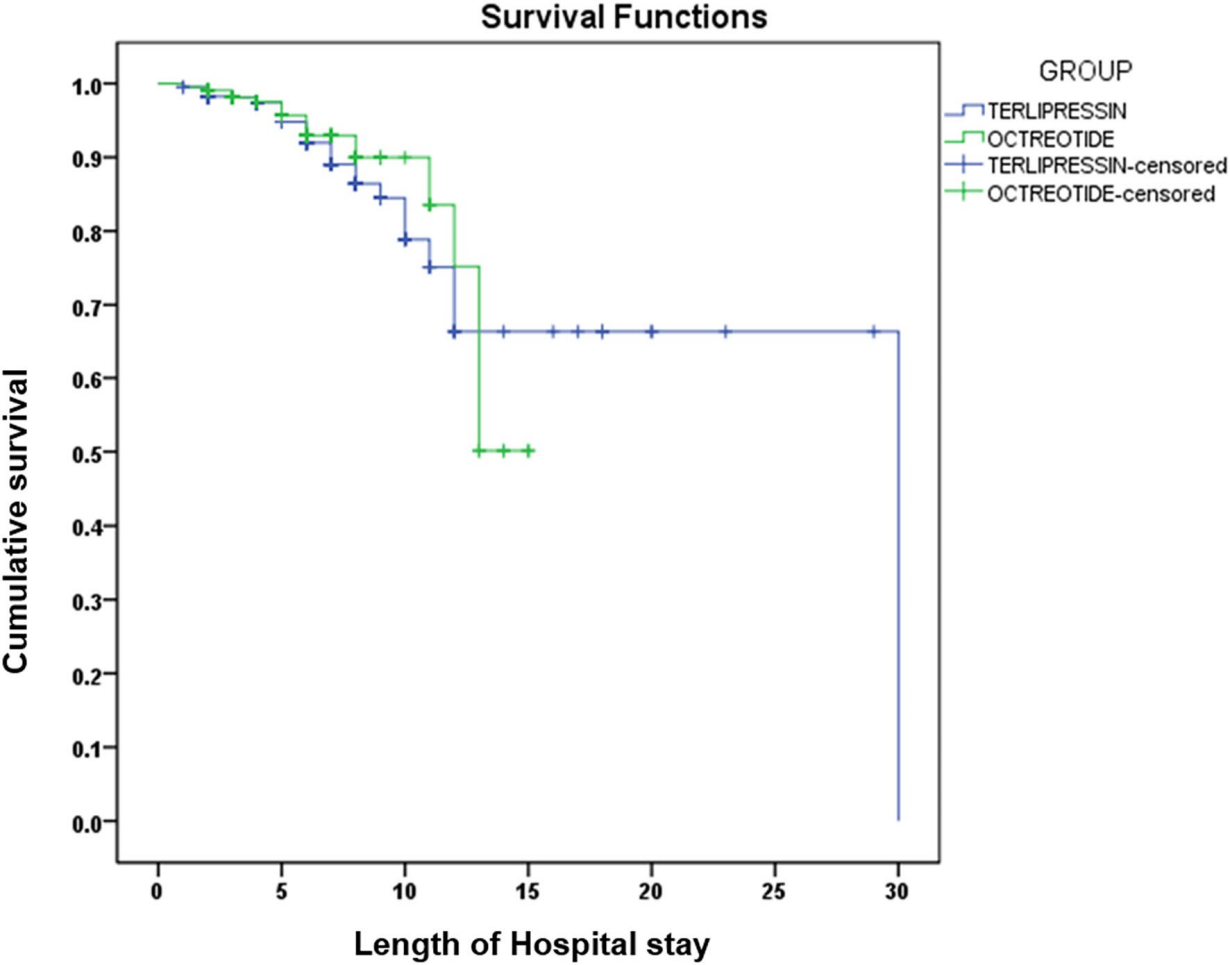
Log rank curve depicting the difference in the length of hospital stay between terlipressin and octreotide groups.

On multiple regression analysis, cardiac events during hospital stay (OR: 3.26), presence of portosystemic encephalopathy at presentation (OR: 3.06), and administration of octreotide (OR: 1.80) were identified as independent risk factors for increased length of hospital stay. Administration of octreotide shows a statistically significant increased length of hospital stay but clinically the difference is negligible as seen in Table 3. Moreover, patients who had tachycardia in the ER, and those with elevated bilirubin levels were associated with a slightly increased risk of prolonged hospital stay (Table 3). Achieving control of variceal bleeding after endoscopy led to a decreased length of hospital stay (OR:0.33) as represented in our log rank test in Fig. 2**.**

**Table 3:** Multiple regression analysis-factors associated with increased length of hospital stay.

	Unadjusted odd ratio (95% CI)	Adjusted odd ratio (95% CI)	*p* value of adjusted odd ratio
Age, years	1.04 (0.99–1.02)	–	
Child class		–	
A	1		
B	1.73 (0.76–3.94)		
C	1.87 (1.16–3.0)		
Hb at presentation (g/dl)	1.05 (0.97–1.13)		
Pulse in ER (beats/min)	1.008 (1.0–1.01)	1.01 (1.0–1.01)	0.04
Oxygen saturation %	0.95 (0.90–1.01)	–	
Serum creatinine (mg/dl)	1.30 (1.10–1.53)	–	
SGPT	1.00 (1.0–1.006)	–	
Total bilirubin (mg/dl)	1.07 (1.04–1.10)	1.05 (1.02–1.09)	0.002
Serum albumin	0.58 (0.43–0.78)	–	
Prothrombin time (s)	1.007 (0.99–1.02)	–	
Control of variceal bleeding after EGD			
No			
Yes	1 0.27 (0.17–0.42)	1 0.33 (0.20–0.56)	< 0.001
Cardiac effects during hospital stay			
No			
Yes	1 5.35 (2.55–11.24)	1 3.26 (1.34–7.95)	0.009
NG tube		–	
No	1		
Yes	1.32 (0.94–1.83)		
PSE at presentation			
No	1	1	
Yes	4.13 (2.87–5.96)	3.06 (2.00–4.67)	< 0.001
Group			
Terlipressin	1	1	
Octreotide	1.73 (1.22–2.43)	1.80 (1.20–2.69)	0.004

### Factors affecting mortality at 6 weeks

On multiple regression analysis, cardiac events during hospital stay (OR: 11.22), presence of Porto-systemic encephalopathy (OR: 3.79), and elevated bilirubin levels at the time of presentation were found to be independent risk factors for increased 6 weeks mortality. On the-other hand, control of variceal bleeding after endoscopy was a protective factor in terms of mortality (OR: 0.09) (Table 4). The use of terlipressin or octreotide was not found to be associated with any significant impact on mortality.

**Table 4:** Multiple regression analysis of factors predicting mortality.

	Adjusted odd ratio (95% CI)	*p* value
Total bilirubin (mg/dl)	1.05 (1.008–1.10)	0.02
Serum albumin	0.36 (0.17–0.77)	0.009
Control of variceal bleeding after EGD		
No	1	
Yes	0.09 (0.04–0.21)	< 0.001
Cardiac effects during hospital stay		
No	1	
Yes	11.22 (3.60–35.0)	< 0.001
PSE at presentation		
No	1	
Yes	3.79 (1.65–8.69)	0.002

We further investigated on renal insufficiency and its effect on death in hospital within the two groups and segregation point was kept at creatinine level of 1.5 mg/dl. Although we have 842 patients, however, in 5 patients creatinine was not performed or the data is missing. Therefore, in the analysis there are 837 patients. Terlipressin accounted for 108 of 621 and octreotide accounted for 57 of 216 (Table 5). Although on subgroup analysis we found statistically insignificant distinctness in mean number of death in hospital who were receiving Terlipressin and Octreotide and had their creatinine above 1.5 mg/dl (Table 6). Out of this total count, eight patients dies from the terlipressin group after 2 months, of which five patients had their creatinine above 1.5 mg/dl. It is necessary to mention that post two-month subgroup analysis for death occuring in these patients, could not be conducted, as there were no reported deaths in hospital and overall as well in the octreotide group.

**Table 5:** Stratification of patients having renal insufficiency in terlipressin and octreotide receiving groups.

Treatment	Level of creatine			P-value
	> 1.5	< 1.5	Total	
Terlipressin	108	513	621	0.004*
Octreotide	57	159	216	
Total	165	672	837	

*P < 0.05 hence statisitcally significant.

**Table 6:** Subgroup analysis of patients having renal insufficiency (creatinine > 1.5 mg/dl) resulting in mortality.

Treatment	Cr > 1.5	Mean number of death in hospital				P-value**
	N	Mean	SD*	Total		
Terlipressin	108	1.17	0.38	1.24	1.1	0.2312
Octreotide	57	1.11	0.31	1.18	1.02	
Total	165	165	672	1.21	1.09	

*p > 0.05 hence insignificant.

## Discussion

This study is a real-time evaluation of a large data set of patients, who received either terlipressin or octreotide, as an adjunct to endoscopic intervention in patients with EVB.

The rationale for the use of vasoactive drugs was to produce splanchnic vasoconstriction and reduce inflow of portal blood and portal pressure^9^. Overall, a higher proportion of patients in our center received terlipressin relative to octreotide, since terlipressin has been shown to effectively control EVB, decrease the requirement of blood transfusion and increase survival^4^. There have been several trials that analyzed terlipressin and octreotide on a head-to-head efficacy basis. Early data suggested a greater effect of terlipressin over octreotide at reducing variceal pressure, and that prompted researchers to recommend terlipressin as the first choice, followed by octreotide or somatostatin as the second choice agents^10,11^. However, many other studies that compared the different vasoactive drugs as monotherapy found no significant difference in the treatment response in patients with variceal bleeding^12^.

Studies have acknowledged the importance of Child–Pugh classification as being an independent mortality-predicting factor in EVB but no differences in mortality were noted between the two treatment groups when the analysis was performed based on the Child–Pugh classification^13^. Majority of our study subjects were males. Most of the patients had advanced liver disease with Child–Pugh Class C. It is known that terlipressin which is a potent vasoconstrictor, can provoke severe dysrhythmias and ischemic complications, and hence it should be used with caution in patients with preexisting ischemic heart or cerebral disease^14^.

We found that there was no significant difference in achieving control of bleeding after endoscopy for the two treatment groups. Several meta-analyses support this notion and neither of the two drugs is preferred over the other in achieving early control of bleeding. Zou et al. in their network meta-analysis conducted in 2019, consisting of 2187 patients found no statistically significant difference with respect to initial control of bleeding^15^. Another meta-analysis conducted in 2012 with data from 1978 to 2008 consisting of 3111 patients with 30 trials failed to prefer any one of the vasoactive drugs over the other over the efficacy of control of bleeding^16^. Similar findings were also reported by Seo et al. in their randomized control trial conducted in 2014^12^. Our present series of large data sets also observed that there were no significant differences in terlipressin and octreotide when used as adjuvant therapy in the management of control of variceal bleeding. This study is also in line with similar findings in our previous randomized controlled trial where we randomly gave terlipressin or octreotide to 164 patients in each arm.

In the present series of patients with variceal bleeding, we found that octreotide was associated with a slightly increased length of hospital stay when compared to terlipressin (OR: 1.59) Our previous study conducted in 2009 also had similar findings^17^. On the contrary, Corley et al. reported a 47% lower risk of complications with Octreotide than with Terlipressin, and a 69% lower risk of presenting major complications, hence shorter hospital stays^18^.

Individual factors that could likely prolong hospital stay were analyzed in our study. In a Cox regression analysis, factors including high pulse, raised bilirubin levels, cardiac events during hospital stay, and presence of portosystemic encephalopathy (PSE) at presentation were all predictors of increased length of hospital stay. Increased pulse in the emergency was associated with an increase in hospital stay. Increased total bilirubin and PSE at presentation were found to be linked with an increased hospital stay. These are well-documented and known facts as both signify the extent of disease progression that can later influence the longevity of its management. Jha et al. state in their terlipressin-focused study that median total bilirubin played a role in the failure of treatment via terlipressin and also influenced early rebleeding^19^. D’Amico et al. also stated that bilirubin levels and the presence of encephalopathy are considered to be independent factors predictive of re-bleeding and mortality, which further supports our study findings^20^.

Six-week mortality was assessed in which cardiac events during hospital stay, PSE, control of variceal bleeding after EGD, total bilirubin, and serum albumin were found to be independent factors influencing/ predicting mortality. Low albumin can increase the chances of mortality. Hence it is combined with terlipressin or octreotide while treating EVB^21,22^.

Control of variceal bleeding after EGD was associated with a decreased length of hospital stay. A review article in 2015 analyzed five comprehensive studies of randomized control trials that accounted for rebleeding in patients treated with vasopressin/terlipressin or somatostatin/octreotide and found no difference in the rebleeding rate between patients treated with either^2^. Therefore our study findings could be attributable to the fact there were more patients with higher MELD scores in our octreotide group and hence more sick patients and relatively longer hospital stay. Moreover, the differences in the hospital stay are practically less than a day which in our opinion is not clinically important.

Our study also suggests no significant difference in mortality between groups that received terlipressin or octreotide. This is consistent with recent trials comparing the two drugs^12^.

A Cochrane systematic review conducted in 2003 recommended the use of Terlipressin as the vasoactive drug of choice in variceal bleeding, as it had a 34% relative risk reduction in mortality^23^. A recent meta-analysis showed that there was no significant difference in mortality with the use of different vasoactive drugs, but terlipressin was observed to have a better response to other outcomes such as rebleeding and requirement for blood transfusion^24^. Another meta-analysis consisting of over 2000 patients concluded that octreotide was the safest vasoactive drug, with the lowest risk of adverse events (9.1%) and serious adverse events (0.0%) compared to other drugs^15^.

Achieving early control of variceal bleeding leads to decreased mortality (OR: 0.09). Although EVB-related mortality at 6 weeks has decreased to approximately 10–20% in the last decades, it is still significant^25,26^. Patients presenting with rebleeding were found to have higher cases of mortality^2^. The rebleeding risk in patients who survive an episode of EVB is very high; 63% at 1 to 2 years with a mortality of 33%^27^.

While comparing the outcomes of the two drugs for EVB, it was observed that the differences between cardiac events during hospital stay, achieving control of variceal bleeding, number of packed cell transfusions received, length of hospital stay, and 6-weeks mortality were found to be not statistically significant. When looking for cardiac events we accounted for acute coronary syndrome and arrhythmias requiring intervention. Azam et al. found relevant differences in their study with cardiac effects in comparison to terlipressin and octreotide. They shared an isolated event of a patient with no previous cardiac history who was initiated on terlipressin but developed ECG changes and was shifted to octreotide^28^.

Cardiac events during hospital stay proved to be a major mortality-predicting factor having an adjusted OR of 11.22 (3.60–35.0). Having pre-existing cardiac conditions puts one at risk of developing further cardiac conditions during stressful body states such as EVB. Although our study did not specify differences in our drug groups playing a role in predicting mortality, a recent meta-analysis consisting of 21 RCTs with a total of 2431 patients identified the risk to be similar between the two drug groups^13^.

Some of the limitations in our study include a relatively larger number of patients in the terlipressin group compared to octreotide, which may influence the comparison. This also reflects a potential selection bias as the allocation of medicine was at the physician’s discretion. The retrospective nature of this study cannot take care of some other unknown confounders. Moreover, hemodynamic studies/hepatic flow studies (Hepatic Venous Pressure Gradient and Portal venous flow) were not done which could have contributed to providing factors of importance while comparing the control of bleeding and survival outcome between groups.

Our study's strength lies in the fact that the results of the present series are based upon the analysis of a large data set, hence the comparison between a large number of patients is meaningful. This study also does not have any observer bias as physicians and patients were not aware of any ongoing study, hence ruling out the possibility of influencing and modulating their treatment behavior, decision-making, and documentation.

Overall, our study did not find much difference in the use of terlipressin and octreotide in terms of mortality. There is some statistically significant difference in terms of prolonged hospital stay but, in our opinion, this has no clinical significance. Moreover, several other facts have been reinforced in our study which include factors such as low hemoglobin, high Child score, high bilirubin levels, and low albumin lead to higher morbidity and mortality in patients who develop esophageal variceal bleeding. Our study is adding and highlighting the facts as eluded in existing literature. Future studies can focus on better understanding other independent and dependant factors and their effects on morbidity and mortality in such presentations. It would advisable to also ponder on a more effective, more comprehensive and cost effective analysis is required in future studies.

In conclusion, both agents have similar outcomes in terms of control of bleeding, hospital stay, mortality. There is no difference in the cardiac effects outcome in the groups. Both agents are safe to use in variceal bleeding management.

## Data Availability

All data generated or analyzed during this study are included in this published article. For further assistance, the corresponding author can be reached at shahab.abid@aku.edu.
